# Improving Access to Mental Health Care and Psychosocial Support within a Fragile Context: A Case Study from Afghanistan

**DOI:** 10.1371/journal.pmed.1001225

**Published:** 2012-05-29

**Authors:** Peter Ventevogel, Willem van de Put, Hafizullah Faiz, Bibiane van Mierlo, Majeed Siddiqi, Ivan H. Komproe

**Affiliations:** 1 Department of Research and Development, HealthNet TPO, Amsterdam, The Netherlands; 2 Mental Health Project, International Medical Corps, Kabul, Afghanistan; 3 Country Office Afghanistan, HealthNet TPO, Kabul, Afghanistan; 4 Department of Social and Behavioural Sciences, Utrecht University, Utrecht, The Netherlands

## Abstract

As one article in a series on Global Mental Health Practice, Peter Ventevogel and colleagues provide a case study of their efforts to integrate brief, practice-oriented mental health training into the Afghanistan health care system at a time when the system was being rebuilt from scratch.

**Summary Points**

- After the fall of the Taliban, the rebuilding of the Afghan health care system, from scratch, provided opportunities to integrate mental health into basic health services through the use of funds that became available during this complex humanitarian emergency.
- Practice-oriented mental health trainings for general health workers and ongoing clinical supervision in the basic health care system led to substantially increased demand for and access to basic mental health care services.
- Treatment of mental disorders within the health care system needs to be accompanied by a community-based approach that focuses on psychosocial problems.
- Addressing service delivery needs in a fragile state has to be accompanied by capacity building and policy development in order to foster structural changes within the health care system.

*This case study is part of the* PLoS Medicine *series on Global Mental Health Practice.*

## Background

By the fall of 2001, when an international military coalition intervened in Afghanistan to oust the Taliban from power, the Afghan people had already survived 23 years of armed conflict. The national health situation was disastrous, with high rates of morbidity and mortality for reproductive health conditions, childhood illnesses, and infectious disorders [1]. Within this context, epidemiological surveys in Afghanistan found high rates of self-reported symptoms of depression, anxiety, and posttraumatic stress, particularly among women and girls [2]–[4]. Much of the psychological distress experienced by the Afghan people could be understood as the result of daily stress endured within the context of ongoing adversity and failing institutions [5],[6]. As a result of these failures, the roles of local social structures, such as family and community, were, and remain, of critical importance to preserve psychosocial wellbeing [7]. Furthermore, there were no reliable data on the prevalence of severe mental health problems in Afghanistan, but, in general, it is estimated that the prevalence of severe mental disorder may rise 50% after emergencies [8].

The health care system in Afghanistan had been completely shattered; there were severe shortages of health care staff, supplies, and infrastructure. Furthermore, the *organisation* of the health care system itself was largely insufficient, plagued by weak coordination and management of service delivery, ineffective policy-making mechanisms, and insufficient information for appropriate health policy planning. All of these features mean that Afghanistan is an example of a “fragile state,” characterised by a government that lacks the capacity to provide core services and basic security to its population. Improving health care within fragile states needs to focus on quick and tangible improvement of services to the people, while also addressing long-term development and strengthening of the health care system [9].

In a situation of overwhelming needs and scarce resources, the mental health care system was disproportionally affected. Afghanistan's formal mental health services were limited to a few services in regional hospitals, as the country's only psychiatric hospital had been destroyed. In 2001, with an estimated population of over 25 million people, there were only two psychiatrists, and no formally qualified psychiatric nurses or clinical psychologists in the country. There was no department responsible for mental health care in the Ministry of Public Health (MoPH) [10].

Within this context, it is important to enable the health care system to address at least some basic needs of people with mental disorders, and to strengthen the capacity of families and communities to address psychosocial distress [11],[12]. This paper reports the experiences of an international nongovernmental organisation (NGO), HealthNet TPO, in Nangarhar (one of Afghanistan's 34 provinces). We describe our work attempting to develop three interrelated features of creating a sustainable system for mental health and psychosocial support within a fragile setting: 1) integration of services for some mental disorders within the general health care system; 2) community-based activities to strengthen self-help initiatives and social action; and 3) support for mental health policy development at the central, government level.

## Integrating Mental Health Care into Basic Health Services

After the fall of the Taliban in 2001, health service delivery was contracted out to NGOs by the government and international donors [13]. The aim was to rapidly scale-up health services, utilising well-described interventions, and replace fragmented and uncoordinated services with an acceptable level of basic services countrywide. The Basic Package of Health Services (BPHS) describes minimum interventions to be provided at various levels of the general health care system. Mental health care was initially included in the BPHS (2003), but these interventions were not well described. Donors doubted the feasibility of including them within the core package and any organizations who wanted to integrate mental health care into the basic services had to develop their own methods and tools. Integrating mental health services into primary care is the most viable way of ensuring that people with mental disorders receive basic mental health care. However, there is no single best practice model that can be followed in all countries [14]. Longitudinal data from Pakistan demonstrated that integrating mental health into primary care services may strengthen the functionality of the primary health care system as a whole [15].

In the eastern province of Nangarhar (population 1.38 million [16]), provision of health care services was contracted out to HealthNet TPO. The needs assessment included focus group discussions to explore local concepts of mental illness and health-seeking behaviour. In consultation with provincial health authorities and local service providers, the programme prioritised common mental disorders (which included depressive and anxiety disorders), severe mental disorders (such as psychosis), and epilepsy. For each of the levels of the health care system staff were trained in identification and management of priority conditions, using locally developed modules [17]:

1. *Health posts:* Community health workers (CHWs) typically have 3 months of training in health issues, offer limited curative care, and provide health education and referrals. The mental health care training aspect consisted of a 3-day course focused on identification of people with possible mental health issues within the community, and follow-up of treatment adherence of patients with chronic, disabling mental disorders.
2. *Basic health centres and comprehensive health centres*: These are small health facilities covering a population of 15,000 to 30,000 people (basic health centre, BHC) to 30,000 to 60,000 people (comprehensive health centre, CHC), staffed by physicians, nurses and midwives. After two series of 10-day trainings, health workers learned to identify the priority disorders and formulate a treatment plan. Training strongly focused on improving clinical competence using demonstration videos, role play, and inviting patients to contribute to the training. Physicians received additional training in the appropriate prescription of psychotropic medication and nurses and midwives received additional training in basic psychosocial interventions, such as psycho-education for patients and family members.
3. *District hospitals:* Outpatient and inpatient services were made accessible for patients with mental health problems. Each hospital had a full-time physician trained in mental health care. The 2-month training included an internship in a psychiatric department of an academic hospital in Pakistan. District hospitals also employed a psychosocial worker to provide psycho-education and organise support groups.

The first mental health trainings were given at the end of 2002, in six rural districts (the “Shinwar cluster”), with an estimated 339,053 inhabitants. During this *development phase*, training materials and methodology were developed, tested, and adapted. Medication supply occurred using existent supply lines and included all psychotropic drugs from the World Health Organization (WHO) Model List of Essential Medicines, with the exception of lithium carbonate and methadone [18]. In the *scale-up phase*, from 2005 to 2008, the programme was rolled out over the entire province. An important element in this scaling-up was a 2-month training for core staff to strengthen their clinical skills and enable them, in turn, to train and supervise general health care staff. Supervisors of the programme, general physicians and nurses trained in mental health, visited the trained health staff at least once a month. Gradually, mental health care has been integrated into the tasks of the general supervision system, through joint supervisions from a general health supervisor and a mental health supervisor. From 2009 onwards, there was no specific funding for mental health programmes; therefore, all activities, including the training of new staff and supervision and provision of psychotropic drugs, have been integrated within the general health budgets (*maintenance phase*).

## Strengthening Community Care and Resilience

Community actors can play a critical role in achieving better outcomes in the field of mental health care and psychosocial wellbeing. For example, there is evidence from India that community mobilisation through participatory women's groups can reduce mild and moderate forms of depression, and provide a powerful addition to health-worker-led interventions [19]. For these reasons, the programme developed activities to strengthen community resilience, and focused on women, due to the reports of high levels of mental distress [20]. Activities included i) community psycho-education in health facilities, mosques, and houses of community leaders, which provided large numbers of people with information about psychosocial and mental health problems, as well as ways to cope; ii) *workshops* for village volunteers, or community health workers, about topics such as: grief, drug use, child rearing, domestic violence, and mental health issues; iii) *support groups*, in which people came together to tell their story, or discuss a problem, with the specific aim of receiving support and/or learning from other participants; and iv) individual case management through *supportive counselling* [21].

## Policy Support

In order to build a health care system within a fragile context, it is also important to invest in strengthening policy making [22]. HealthNet TPO has been an active partner of the MoPH. In 2003 and 2006, the NGO assisted the MoPH in organising national conferences on improving the mental health care system. In 2005, with the financial and technical support of HealthNet TPO, a Mental Health Department was established in the MoPH. This enabled the ministry to take a leading role in policy development and service coordination.

## Results

### Improved Capacity of Service Providers

Since the programme's initiation in 2002, 334 doctors, 275 nurses and midwives, and 931 CHWs have received basic mental health training in Nangarhar. The training covered all 592 health posts, all 39 BHCs, 17 CHCs, and the three district hospitals. The training has enhanced the capacity of health care staff to identify and help people with mental disorders, as is illustrated in Box 1.

**Box 1. A Depressed Farmer**

Rahimullah is a 31-year-old farmer in a mountainous district of Nangarhar province, about 100 kilometres (km) from the provincial capital, Jalalabad. He is responsible for supporting a wife and six children. He presented to a local, private physician with pains in his back, waist, and arms, a burning sensation in his hands and shoulders, and “heaviness” after eating. Additionally, he reported problems with weight loss, sleeping, chronic fatigue, loss of appetite, and a very low to absent libido. He felt overwhelmed with hopelessness, was unable to work, and rarely left home. As there had been no recent problems, nor a recent death in the family, Rahimullah could shed no light on the origin of his symptoms and was prescribed antimalarials.

However, the symptoms persisted, and over the course of a year, he travelled to a variety of physicians in Jalalabad and Peshawar (Pakistan), 200 km away. He submitted to repeated tests for blood, urine, and stool, as well as seven abdominal, kidney, and pelvic ultrasonographic scans. All results were normal, with the exception of a moderately elevated alanine aminotransferase. He was prescribed a huge variety of pharmaceuticals, including antihelmintics, painkillers, antibiotics, and sedatives. The tests and pharmaceutical treatments (in total) cost more than 30,000 Afghani (US$626), or the equivalent of six months of wages.

His symptoms did not decrease as a result of any of these treatments. One year after his symptoms had first manifested, Rahimullah was seen by a local community health worker (CHW) who was able to identify mental health disorders as a result of a 3-day training. The CHW recommended the basic health centre (5 km away), whose staff had also been trained in the diagnosis and treatment of mental health disorders. There, the physician diagnosed a depressive disorder and prescribed 75 mg amitriptyline per day. Additionally, during regular contact with the nurse, he was advised to begin with a few easy tasks each day, and to gradually return to work. After a few weeks, Rahimullah's sleep had improved dramatically, and both the sense of hopelessness and body pains had decreased. A few months later, he resumed work as a farmer.

*This example illustrates how the integration of mental health into primary health care can lead to services that are cheap, simple, and tailored to local needs. It also illustrates the importance of an integrated team of trained health workers: the physician, nurse, and CHW each have a critical, but different, role.*

The person described in Box 1 (named with a pseudonym) has given informed consent (as outlined in the PLoS consent form) to publication of their case details.

### Increased Service Utilisation

**Figure 1:**
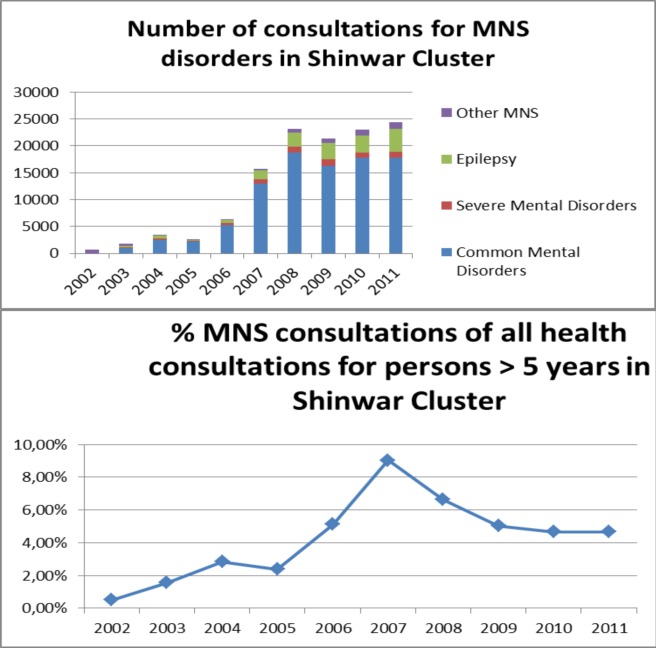
Annual consultations for CMDs, SMDs, and epilepsy and their percentage of all health consultations in the Shinwar cluster (2002–2011).

Since 2002, the number of consultations for mental, neurological, and substance use (MNS) disorders has increased. Figure 1 presents longitudinal data of diagnoses for common mental disorders (CMDs), severe mental disorders (SMDs), epilepsy, and other MNS disorders in patients in the primary health care facilities of the Shinwar cluster. The absolute number of consultations for MNS disorders increased from 659 in 2002 (before the programme started) to well over 3,000 by the end of the development phase (2004). During the scale-up phase, which began at the end of 2005, the numbers of consultations for MNS disorders increased significantly, to over 20,000. During the maintenance phase (from 2009 onwards), the number of consultations for MNS disorders remained stable, but the percentage of such consultations of all health consultations decreased because of contextual changes in the overall health care system, such as concerted efforts to increase the utilisation of reproductive and child health services. From 2008 on, the basic health care system was strongly strengthened, leading to an increase in overall numbers of consultations. Among people newly diagnosed with an MNS disorder, most were diagnosed with CMD (83.2%), followed by epilepsy (8.9%) and SMD (2.7%). The category “other MNS disorders” (including severe learning disabilities and opium addiction) constituted 5.2% of the total new MNS disorders. Persons diagnosed with CMD had, on average, 2.0 annual visits, while those with chronic conditions had a higher number of annual visits: 3.9 and 3.3 for SMD and epilepsy, respectively. Of those patients diagnosed with CMD, 71.1% were female, for SMD the percentage of females was 43.3%, and for epilepsy, 51.8%.

### Involving Communities

In Nangarhar, more than 500 CHWs and 300 teachers were trained in stress management and (domestic) violence related issues. Support groups were organised on a weekly basis to empower women towards an increased autonomy. People presenting with mental health problems received supportive counselling sessions, or were referred to the health care services. It was shown that community-based activities can provide powerful ways to address mild forms of common mental disorders, as illustrated in Box 2.

**Box 2. Support Group on Violence within the Family**

Psychosocial workers from HealthNet TPO invited the women of Mohmandara, a village in the Nangarhar Province (close to the border with Pakistan), to participate in a support group in 2005. Almost all women from the village wanted to participate, specifically to discuss “feelings of sadness” and “worrying too much.” From the first group's first session it became clear that “feelings of sadness” were often a local euphemism to express tension and violence within the family. Additionally, according to the participants, spouses were not the only violent perpetrators; mothers-in-law and sisters-in-law often encouraged the repression of their sons' and brothers' wives. These women were perceived, by the village women, as almost as “evil” as the male perpetrators. All members of the group agreed that it was unthinkable to involve the police, or other outsiders, in order to stop the violence. Therefore, other solutions had to be found. The village women shared their experiences and ideas, over several sessions, to find culturally appropriate solutions. Several concrete solutions were discussed, for example, involving a close relative who could act as a mediator. Improving “communication skills” through role-play was another way to empower the group, as well as teaching the women effective ways to settle disputes. As all the participants of the group agreed that violence within a family was often the result of “stress” due to unemployment and poverty, practical solutions were generated to improve their economic status. The facilitator of the support group, Farida (a pseudonym), a local woman trained by HealthNet TPO, connected the group to a local NGO with income generating activities. The group raised some money to purchase a small flock of chickens. The eggs were then sold on the market, in order to supplement incomes.

*This example illustrates how a support group can evolve over time and can become a self-help group focusing on income generation, as well as providing improved psychosocial functioning.*

The persons described in Box 2 have given informed consent (as outlined in the PLoS consent form) to publication of their case details.

### Mental Health Policy Development

Only with the insistence of the Afghan MoPH, and after NGO projects such as the one described in this paper had demonstrated its feasibility, did donors finally agree to fund mental health care interventions as part of the BPHS [23]. In 2008, a technical working group of the Ministry, WHO, and NGOs including HealthNet TPO produced a full range of mental health training manuals for each type of health worker. In the 2010 revision of the BPHS, the mental health components became stronger, and in 2010, the MoPH endorsed a 5-year National Mental Health Strategy.

## Barriers to Access

A challenge in the expansion of health care, within extremely under resourced settings, is that the rapid increase of service delivery is usually valued over long-term integrated systems development. This carries the risk of creating a relatively large coverage of services with low quality and limited sustainability [24]. In Afghanistan, while the number of patients per health facility has greatly increased, the time spent per health worker per patient did not. In fact, it has shown a decrease, with 80% of patients receiving fewer than 10 minutes per consultation [25]. The pressure to see more patients makes it difficult for health workers to use psychosocial interventions that require time. Also, integrating mental health care into primary care carries the inherent danger of framing context-generated distress as a mental disorder [26]. Medical staff have the tendency to use a “medical” model when solving problems, and to focus on the prescription of drugs. Often, there is an expectation from both the health worker and patient that drugs are to be prescribed during a consultation. When psychosocial aspects of treatment are given less emphasis, particularly with common mental disorders, there is a risk that treatment by medical staff concentrates on the biological (medication). Therefore, for people with CMD, it is important that support in community settings is strengthened and that health providers are trained not to prescribe antidepressant medication for mild forms of depressive disorder [27].

In general, rapidly expanding access to care for a mental disorder within the basic health care system mostly benefits those patients who do not need specialised or long-term care. There is an obvious need to strengthen home-based care for people with chronic, disabling disorders in order to improve therapy compliance and to integrate mental health care into the secondary health care system, particularly the provincial hospitals. In order to respond to that need, HealthNet TPO has opened a 20 bed psychiatric, inpatient unit in the provincial hospital.

## Looking to the Future

The experience in Nangarhar shows that, even within a fragile and resource poor context, it is possible to develop integrated services for mental health and psychosocial support, to rapidly cover an area of more than a million people. It is important to use funds available during a humanitarian emergency to pursue lasting improvements in the health care system [28]. There is an urgent need to develop a system of routine outcome measuring tools that includes both symptom reduction and improvement of social functioning. It is challenging to develop context-specific and low-cost outcome measures, but recent evidence for child psychosocial programmes in post conflict areas demonstrates that it can be done [29]. People with a limited background in mental health care can deliver integrated services, once their tasks are integrated within a system of care that includes focused, competency-based trainings, regular supervision, and refresher training [30]. It is important to strengthen the psychosocial elements of treatment within the health care system, and to ensure that the social context in which the symptoms occur and are maintained, are considered in the treatment plans of health care providers. The most recent version of the BPHS includes the addition of psychosocial counsellors at the district hospitals and comprehensive health centres. Preliminary evidence on the effectiveness of adding psychosocial counselling in primary health care settings in Afghanistan is encouraging [31] Apart from health system–based interventions, the authors have learned the importance of addressing psychosocial problems through activities outside the formal health care sector to strengthen self-help and foster resilience.

## Supporting Information

Alternative Language Abstract S1 Translation of the Summary Points into Dari by Hafizullah Faiz.
(PDF)

Alternative Language Abstract S2 Translation of the Summary Points into Pashto by Hafizullah Faiz.
(PDF)

## Abbreviations

BHC: basic health centre
BPHS: Basic Package of Health Services
CHC: comprehensive health centre
CHW: community health worker
CMD: common mental disorder
MNS: mental, neurological, and substance use disorder
MoPH: Ministry of Public Health
NGO: nongovernmental organisation
SMD: severe mental disorder
WHO: World Health Organization
