# Supplementary material for: Improving Access to Mental Health Care and Psychosocial Support within a Fragile Context: A Case Study from Afghanistan
Source: PLoS Med. 2012 May 29;9(5):e1001225. doi: 10.1371/journal.pmed.1001225 (PMC3362640; doi:10.1371/journal.pmed.1001225)
Supplement: Alternative Language Abstract S1 — Translation of the Summary Points into Dari by Hafizullah Faiz. (PDF) [file pmed.1001225.s001.pdf]

بهبود دسترسی به صحت روان و روان اجتماعی در ممالک دارایی شرایط نازک: یک مثال از افغانستان

پیتر وینتو وخیل (Peter Ventevogel)

شبکه صحی (HealthNet TPO) دیپارتمنت تحقیق و انکشاف، امستردام، هالند

ولم وندی پت (Willem van de Put)

شبکه صحی ، امستردام، هالند

حفیظ الله فیض

پروژه صحت روان، هیت طبی بین المللی (International Medical Corps) کابل، افغانستان

بیبیانی ون میرلو (Bibiane van Mierlo)

شبکه صحی (HealthNet TPO) دیپارتمنت تحقیق و انکشاف ، امستردام، هالند

مجید صدیقی

شبکه صحی ، کابل، افغانستان

ایوان کومرو (Ivan H. Komproe)

شبکه صحی (HealthNet TPO) دیپارتمنت تحقیق و انکشاف ، امستردام، هالند

ساینس روش طرز دید و اجتماعی، پو هنتون یو تریخ ، یوتریخ، هالند

موًلف: **پیتر وینتو وخیل**(Peter Ventevogel) peter@peterventevogel.com

خلاصه:

- از ابتدا ،اهیایی مجدد سیستم صحی شرایط رابرایی ادغام صحت روان در خدمات اساسی صحت توسط بودیجه کی برایی کمکهایی عاجل شده بود آماده ساخت
- کورسها یی صحت روان ایجاد شده به اساس ضرورت به کارکنان عمومی صحت ونظارت منظم ازکارهایی آنها دسترسی را به خدمات صحت روانی در مراقبتهایی اولیه زیاد و خوتبر ساخته
- تداویی امراض روانی در سیستم صحت ،درداخل قریه جات ضرورت به پروگرامهایی أگاهی عامه درباره مسایل روان اجتماعی دارند
- برایی اینکه تغیرات در تشکیلات را نکشاف دهیم، چاره یا غم خوردن خدمات در سیستم صحت در مما لک دارایی شرایط نازک باید همراه با ظرفیت سازی باشد.

Translated by Hafizullah Faiz, M.D
