# Supplementary material for: Improving Access to Mental Health Care and Psychosocial Support within a Fragile Context: A Case Study from Afghanistan
Source: PLoS Med. 2012 May 29;9(5):e1001225. doi: 10.1371/journal.pmed.1001225 (PMC3362640; doi:10.1371/journal.pmed.1001225)
Supplement: Alternative Language Abstract S2 — Translation of the Summary Points into Pashto by Hafizullah Faiz. (PDF) [file pmed.1001225.s002.pdf]

رواني روغتيا پاملرني او روحي تولنيزو مرستو ته دلاسرسی ښه والی په نازکو حالاتو لرونکو ملکونو کی: یوه بیلګه له افغانستان څخه

پیتر وینتو وخیل (**Peter Ventevogel**)

د روغتیایی شبکي (**HealthNet TPO**) د دځیرنو او پراختیا دیپارتمنت، امستردام، هالند

ولم وندی پت (**Willem van de Put**)

روغتیایی شبکه، امستردام، هالند

حفیظ الله فیض

روانی روغتیا پروژه، نړیواله طبی ډله (**International Medical Corps**) کابل، افغانستان

بیبیانی ون میرلو (**Bibiane van Mierlo**)

د روغتیایی شبکي(**HealthNet TPO**) دځیرنو او پراختیا دیپارتمنت، امستردام، هالند

مجید صدیقی

روغتیا یی شبکه، کابل، افغانستان

ایوان کومرو (**Ivan H. Komproe**)

د روغتیایی شبکي(HealthNet TPO) د دځیرنو او پراختیا دیپارتمنت، امستردام، هالند

دټولنیز او کړو وړو ساینس، د یو تریخ پو هنتون، یوتریخ، هالند

مؤ لف: **پیتر وینتو وخیل**(Peter Ventevogel) peter@peterventevogel.com

لنډیز:

- له ابتداڅخه ، د افغانستان دروغتیایی سیستم بیا جوړونه ددی لپاره شرایط برا بر کړل چي روانی روغتیا په بنسټیزو روغتیا یی چوپړتیاووکی مدغمه شی په هغه پانګو چي دبشری عاجلو مرستو لپاره ورکړل شوی.
- په ضرورت ولاړه روانی روغتیا روزنیز و کورسونه د عمومی روغتیایی کارکوونکو لپاره او دهغو له کارونو څخه دوامداره نظارت ، په لمړنیو روغتیایی پاملرنو کی روانی روغتیا ته دډیری او ښه لاسرسی لامل ګرځیدلی.
- دروانی ناروغیو درملنه په روغتیایی سیستم کی، په ټولنه کی دروحي ټولنیزستونزو په اړه عامه پوهاوی کارونو ته اړتیا لری.
- ددی لپاره چی د تشکیلاتو تغیراتو ته وده ورکړل شی په روغتیایی سیستم کی دچوپړتیاو وړاندی کوني غم کول په نارامه یا نازکو حالاتو لرونکو ملکونو   کي باید دظرفیتونو دلوړولو سره یوځای وی.

Translated by Hafizullah Faiz, M.D.
